# Supplementary material for: SnoRNA guide activities: real and ambiguous
Source: RNA. 2021 Nov;27(11):1363–73. doi: 10.1261/rna.078916.121 (PMC8522698; doi:10.1261/rna.078916.121)
Supplement: Supplemental Material [file supp_27_11_1363__DC1.html]

SnoRNA guide activities: real and ambiguous — Supplemental Material 

# SnoRNA guide activities: real and ambiguous

## Supplemental Material

- Supplemental\_Fig\_S1.pdf
