# Supplementary material for: SnoRNA guide activities: real and ambiguous
Source: RNA. 2021 Nov;27(11):1363–73. doi: 10.1261/rna.078916.121 (PMC8522698; doi:10.1261/rna.078916.121)
Supplement: Supplemental Material [file supp_078916.121_Supplemental_Fig_S1.pdf]

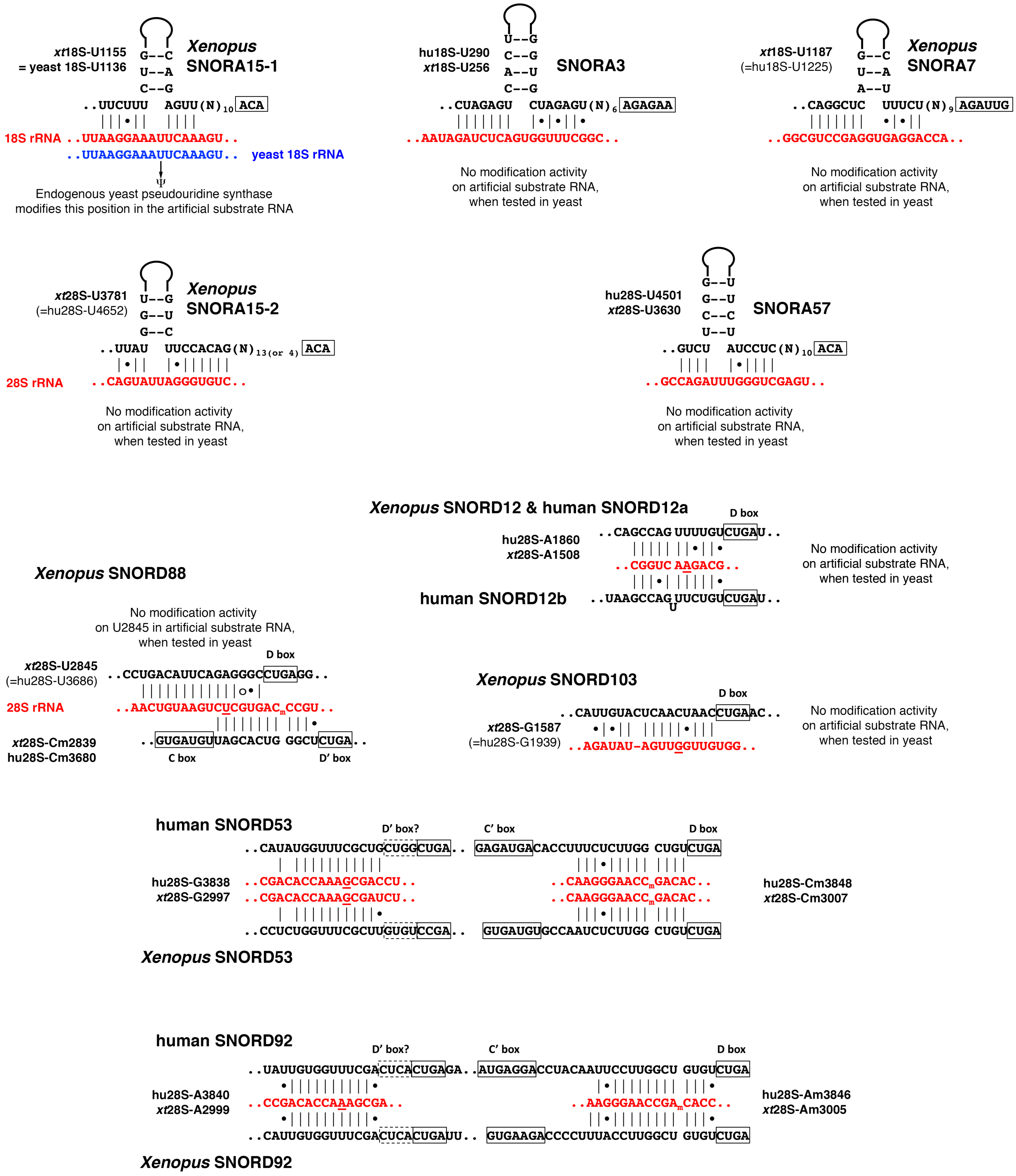

Supplemental Figure S1.

Potential base-pairing of snoRNAs with rRNAs at unmodified positions. rRNA fragments used for artificial substrate RNAs are shown in red. For *Xenopus*-specific ASEs for unmodified positions, equivalent positions in human rRNAs are indicated in parentheses.
